# A Comprehensive Perspective on Intracranial Pressure Monitoring and Individualized Management in Neurocritical Care: Results of a Survey with Global Experts

**DOI:** 10.1007/s12028-024-02008-z

**Published:** 2024-05-29

**Authors:** Sérgio Brasil, Daniel Agustín Godoy, Walter Videtta, Andrés Mariano Rubiano, Davi Solla, Fabio Silvio Taccone, Chiara Robba, Frank Rasulo, Marcel Aries, Peter Smielewski, Geert Meyfroidt, Denise Battaglini, Mohammad I. Hirzallah, Robson Amorim, Gisele Sampaio, Fabiano Moulin, Cristian Deana, Edoardo Picetti, Angelos Kolias, Peter Hutchinson, Gregory W. Hawryluk, Marek Czosnyka, Ronney B. Panerai, Lori A. Shutter, Soojin Park, Carla Rynkowski, Jorge Paranhos, Thiago H. S. Silva, Luiz M. S. Malbouisson, Wellingson S. Paiva

**Affiliations:** 1 Division of Neurosurgery, Department of Neurology, School of Medicine University of São Paulo, Av. Dr. Eneas de Carvalho Aguiar 255, São Paulo, Brazil; 2 Neurointensive Care Unit, Sanatório Pasteur, Catamarca, Argentina; 3 Intensive Care Unit, Hospital Posadas, Buenos Aires, Argentina; 4 Neurosciences and Neurosurgery, Universidad El Bosque, Bogotá, Colombia; 5 Department of Intensive Care, Hôpital Universitaire de Bruxelles, Université Libre de Bruxelles, Brussels, Belgium; 6 Anesthesia and Intensive Care, Scientific Institute for Research, Hospitalization and Healthcare, Policlínico San Martino, Genoa, Italy; 7 Neuroanesthesia, Neurocritical and Postoperative Care, Spedali Civili University Affiliated Hospital of Brescia, Brescia, Italy; 8 Department of Intensive Care, Maastricht University Medical Center, Maastricht, The Netherlands; 9 School of Mental Health and Neurosciences, University Maastricht, Maastricht, The Netherlands; 10 Department of Clinical Neurosciences, Addenbrookes Hospital, University of Cambridge, Cambridge, UK; 11 Department and Laboratory of Intensive Care Medicine, University Hospitals Leuven, Leuven, Belgium; 12 Departments of Neurology, Neurosurgery, and Center for Space Medicine, Baylor College of Medicine, Houston, TX USA; 13 Neurology Department, São Paulo Federal University Medical School, São Paulo, Brazil; 14 Department of Anesthesia and Intensive Care, Health Integrated Agency of Friuli Centrale, Udine, Italy; 15 Department of Anesthesia and Intensive Care, Parma University Hospital, Parma, Italy; 16 University of Cambridge, Cambridge, UK; 17 Cleveland Clinic Neurological Institute, Akron General Hospital, Fairlawn, OH USA; 18 Uniformed Services University, Bethesda, USA; 19 Brain Trauma Foundation, New York, USA; 20 Division of Neurosurgery, Addenbrooke’s Hospital, Cambridge, UK; 21 Cerebral Haemodynamics in Ageing and Stroke Medicine Group, Department of Cardiovascular Sciences, University of Leicester, Leicester, UK; 22 Departments of Critical Care Medicine, Neurology and Neurosurgery, University of Pittsburgh School of Medicine, Pittsburgh, PA USA; 23 Departments of Neurology and Biomedical Informatics, Columbia University Vagelos College of Physicians and Surgeons, New York-Presbyterian Hospital, New York, NY USA; 24 Department of Urgency and Trauma, Medical Faculty, Federal University of Health Sciences of Porto Alegre, Porto Alegre, Brazil; 25 Intensive Care and Neuroemergency, Santa Casa de Misericórdia, São João del Rei, Brazil; 26 Department of Intensive Care, School of Medicine University of São Paulo, São Paulo, Brazil

**Keywords:** Cerebral perfusion pressure, Individualized care, Intracranial pressure, Intracranial pressure waveform, Intracranial compliance, Neurocritical care

## Abstract

**Background:**

Numerous trials have addressed intracranial pressure (ICP) management in neurocritical care. However, identifying its harmful thresholds and controlling ICP remain challenging in terms of improving outcomes. Evidence suggests that an individualized approach is necessary for establishing tolerance limits for ICP, incorporating factors such as ICP waveform (ICPW) or pulse morphology along with additional data provided by other invasive (e.g., brain oximetry) and noninvasive monitoring (NIM) methods (e.g., transcranial Doppler, optic nerve sheath diameter ultrasound, and pupillometry). This study aims to assess current ICP monitoring practices among experienced clinicians and explore whether guidelines should incorporate ancillary parameters from NIM and ICPW in future updates.

**Methods:**

We conducted a survey among experienced professionals involved in researching and managing patients with severe injury across low-middle-income countries (LMICs) and high-income countries (HICs). We sought their insights on ICP monitoring, particularly focusing on the impact of NIM and ICPW in various clinical scenarios.

**Results:**

From October to December 2023, 109 professionals from the Americas and Europe participated in the survey, evenly distributed between LMIC and HIC. When ICP ranged from 22 to 25 mm Hg, 62.3% of respondents were open to considering additional information, such as ICPW and other monitoring techniques, before adjusting therapy intensity levels. Moreover, 77% of respondents were inclined to reassess patients with ICP in the 18–22 mm Hg range, potentially escalating therapy intensity levels with the support of ICPW and NIM. Differences emerged between LMIC and HIC participants, with more LMIC respondents preferring arterial blood pressure transducer leveling at the heart and endorsing the use of NIM techniques and ICPW as ancillary information.

**Conclusions:**

Experienced clinicians tend to personalize ICP management, emphasizing the importance of considering various monitoring techniques. ICPW and noninvasive techniques, particularly in LMIC settings, warrant further exploration and could potentially enhance individualized patient care. The study suggests updating guidelines to include these additional components for a more personalized approach to ICP management.

**Supplementary Information:**

The online version contains supplementary material available at 10.1007/s12028-024-02008-z.

## Introduction

Our understanding of intracranial pressure (ICP), a critical indicator of brain health, is continuously evolving [1], with satisfactory evidence supporting its monitoring as a means of outcome improvement [2, 3]. However, both the safety limits and therapeutic thresholds for managing and treating ICP are still debatable [4, 5], although the current recommendation is based on 22 mm Hg [6]. Several notable trials investigating traumatic brain injury (TBI), whether targeting ICP alone [7] or in combination with brain oximetry [8], employing various therapy strategies, such as decompressive craniectomy [9, 10], hypothermia [11], and others, have highlighted the ongoing need for further advancement in outcome improvement. Possibly, part of this issue may be attributed to attempts to oversimplify ICP as a binary “yes” or “no” phenomenon, neglecting to individualize ICP thresholds based on disease processes or individual variations.

The generation of pressure within the cranium is a dynamic process involving various anatomical structures, such as the brain, cerebrospinal fluid, arterial and venous blood volumes, meninges, and bones. Additionally, pressure in abdominal, thoracic, and cervical cavities also influences intracranial space pressure [12]. Following an acute brain injury, inflammatory cascades and impairment in cerebral physiological components contribute to brain tissue volume expansion (cerebral edema) and elevation of ICP [13, 14]. Moreover, factors such as ventilator-patient desynchrony, improper patient positioning, sedation use, arterial blood pressure (ABP), and volemic management, as well as complications such as ischemia, vasospasm, infection, and secondary hemorrhage, may also lead to intracranial hypertension (IH) development [12].

**Fig. 1:**
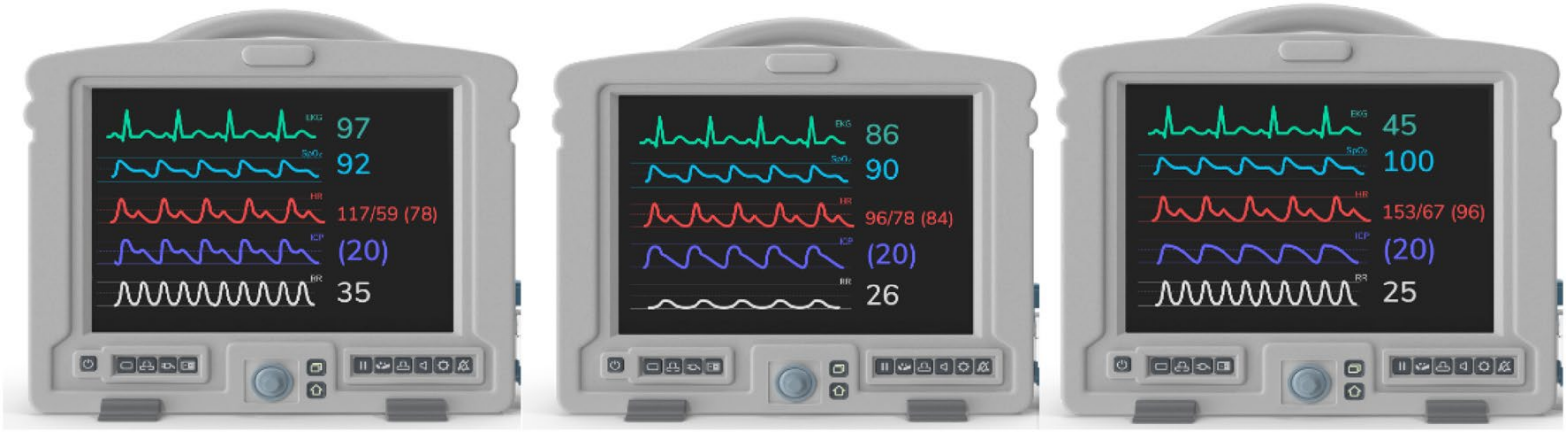
Three different patients with the same ICP value (20 mm Hg) but distinct ICP pulse morphologies (purple waveforms) representing distinct levels of intracranial compliance. *ICP* intracranial pressure (Color figure online)

Among these factors, the intracranial variation of blood volume during each heartbeat is the primary determinant of ICP dynamics, at least on a second-by-second timescale [15]. Beat by beat, ICP pulse slopes can be observed, showing habitual systolic and diastolic phases in dedicated monitors. This pulse morphology can indicate compromised intracranial compliance (ICC) and may predict IH [16–18]. Therefore, absolute ICP numbers alone may not be precise indicators because they do not reflect ICC and may not assist in determining therapy strategy following different IH syndromes [19] and may lead to imprecise cerebral perfusion pressure (CPP) calculation [20]. This underscores the need for additional parameters for guidance. In this context, multimodality neuromonitoring and ICP waveform (ICPW) or pulse morphology emerge as promising options for better differentiation of patients at risk of developing IH crisis [21, 22] (Fig. 1).

The present study is a survey on perceptions regarding ICP monitoring among neurocritical care professionals. The primary objective was to gather opinions on current ICP monitoring practices from professionals with diverse backgrounds, for a discussion on individualizing ICP management using additional monitoring tools, especially ICPW and noninvasive monitoring (NIM). The study aimed to determine whether, from an expert standpoint, ICP management guidelines can consider the inclusion of ancillary physiological parameters derived from NIM and ICPW in future updates. Differences in opinions among professionals from high-income countries (HICs) and low-middle income countries (LMICs) were explored.

## Methods

A cross-sectional survey was developed to assess professional practices and knowledge regarding ICP, CPP, and ICPW. The survey was crafted by a steering committee following the checklist for reporting of survey studies (CROSS) recommendations (Supplemental 1) previously published [23]. A pilot test involving five professionals was conducted, and based on their feedback, the survey was refined before dissemination via email, social media, and medical meetings to eligible participants (details provided in the following section). The survey was hosted on Google Forms for broad accessibility and was available from October 1st to November 30th, 2023. Participation was voluntary and anonymous, with each participant permitted to submit only one response. The online form was designed to automatically conclude once all questions were answered. Additionally, participants were encouraged to share the survey link with other professionals with similar backgrounds who met the participation criteria.

### Survey

The survey is shown in Supplemental 2. It pertained to patients with acute brain injury undergoing invasive ICP monitoring (regardless of an external drain, parenchymal, or other). Other invasive techniques (e.g., brain oximetry, microdialysis) were not considered as routine, but participants could discuss these techniques in the free-text fields. The survey results, which include an analysis of the divergence in opinions between LMIC and HIC clinicians, are presented following the assessment of each field, which were as follows: (1) agreement with the 22 mm Hg threshold to either start or escalate IH therapy intensity levels (TILs) (based on the Seattle International Brain Injury Consensus Conference recommendations [6]), (2) general knowledge on ICPW and its practical relevance, (3) agreement on using NIM and ICPW to support the individualization of ICP judgment in selected cases, and (4) agreement with including these additional parameters in future guideline updates.

### Participants’ Characteristics

Those surveyed should be active practitioners or researchers with ten or more years of experience in the management and/or research in neurocritical care possessing experience in ICP dynamics. Therefore, participants also should have participated in academic production in the scope of the survey. Participants were not necessarily medical doctors but also nurses and other professionals associated with this practice. The survey was composed of 15 questions in English; therefore, English proficiency was an additional prerequisite. The questions allowed a single response and space for providing any additional comment. Exclusively in the case of ancillary tools available in each participant’s health facility, multiple answers were possible.

### Statistical Analysis

A standard descriptive analysis was performed. Variables were presented through absolute and relative frequencies and, when applicable, 95% confidence intervals (CIs) were calculated with the binomial exact method. Inferential exploratory analyses were conducted and, when applicable, the groups were compared using the *χ*^2^ test. LMIC and HIC were defined as the World Bank classification (available at https://datahelpdesk.worldbank.org). There were no missing data. All tests were two-tailed, and final *p* values under 0.05 were considered significant. The analyses were conducted with the Statistical Package for Social Sciences software (IBM SPSS Statistics for Windows, version 24.0; IBM Corp., Armonk, NY).

## Results

### Participants

This survey recruited 109 participants meeting the inclusion criteria, being evenly distributed between HIC and LMIC (55 and 54 participants, respectively). Among the represented institutions, 48 (54%) were from HIC, and 73 (82%) were academic institutions out of a total of 88. The participants consisted of 6 (5%) nurses, 70 (64%) intensivists, 6 (5%) neurologists, 4 (3%) brain physiologists, and 23 (21%) neurosurgeons involved with the care of patients with severe neurological injury.

### Guidelines Adherence and CPP Measurement

**Fig. 2:**
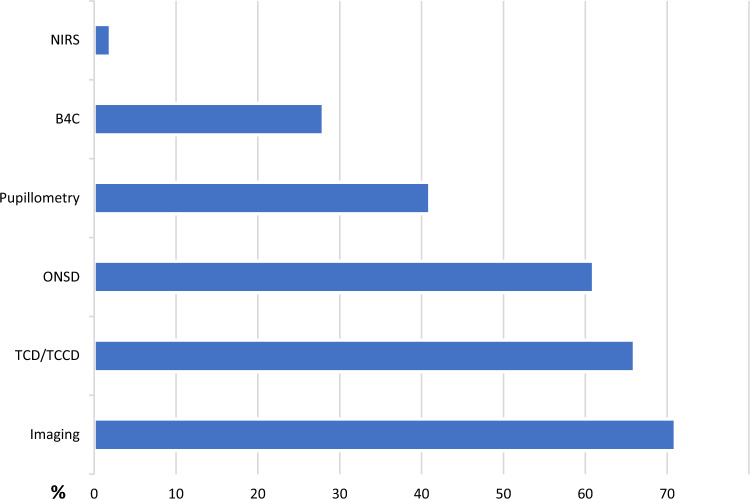
The most used noninvasive techniques to support ICP monitoring among the participants (in percentages of incidence). Data are presented in percentages. *B4C* Brain4Care, *ICP* intracranial pressure, *NIRS* near-infrared spectroscopy, *ONSD* optic nerve sheath diameter, *TCCD* transcranial color-coded duplex, *TCD* transcranial Doppler

The survey revealed a common practice of individualizing target ranges for ICP and CPP, with a frequent reliance on adjunctive NIM techniques to support decisions in the scenario of a patient with acute brain injury with invasive ICP monitoring. Only 30 (27.5%) participants followed 22 mm Hg as the threshold for initiating or escalating TIL. A total of 68 (62.4%) participants were more flexible on cutoff choices, and 11 (10.1%) participants surveyed did not follow the recommended cutoff. Regarding CPP monitoring thresholds, 39 (35.7%) participants pursue the 60–70 mm Hg range, whereas 44 (40.3%) rely on this range when it is supported by ancillary means, such as NIM and imaging, and 26 (23.8%) do not rely on this range. Figure 2 shows the most used NIM techniques among participants. The level with which ABP is zeroed and the transducer positioned, which impacts directly with CPP calculation, is also heterogenous; among the respondents, this level was the heart for 60 (55%) participants, the tragus for 36 (33%) participants, and both levels for 13 (11.9%) participants.

### Relevance of ICPW and NIM

With reference to ICPW monitoring, 71 (65%) participants surveyed considered ICPW fundamental to individualize ICP management, whereas 38 (35%) reported using ICPW in select cases only. The correlation of professionals who consider ICPW fundamental to individualize their patients’ treatment with support to the inclusion of ICPW analysis in future guidelines update was significant (*p* = 0.004). Regarding the use of NIM techniques to support ICP plus CPP monitoring and management individualization, 82 (75.2%) participants agreed to use both NIM and ICP, 23 (21.1%) declared to use NIM occasionally, and only 4 (3.6%) did not see value in using NIM to refine treatment guidance. Likewise, the position of clinicians on behalf of NIM parameters inclusion in future guidelines update was significant (*p* = 0.019).

### Role of ICPW in Different ICP Situations

**Fig. 3:**
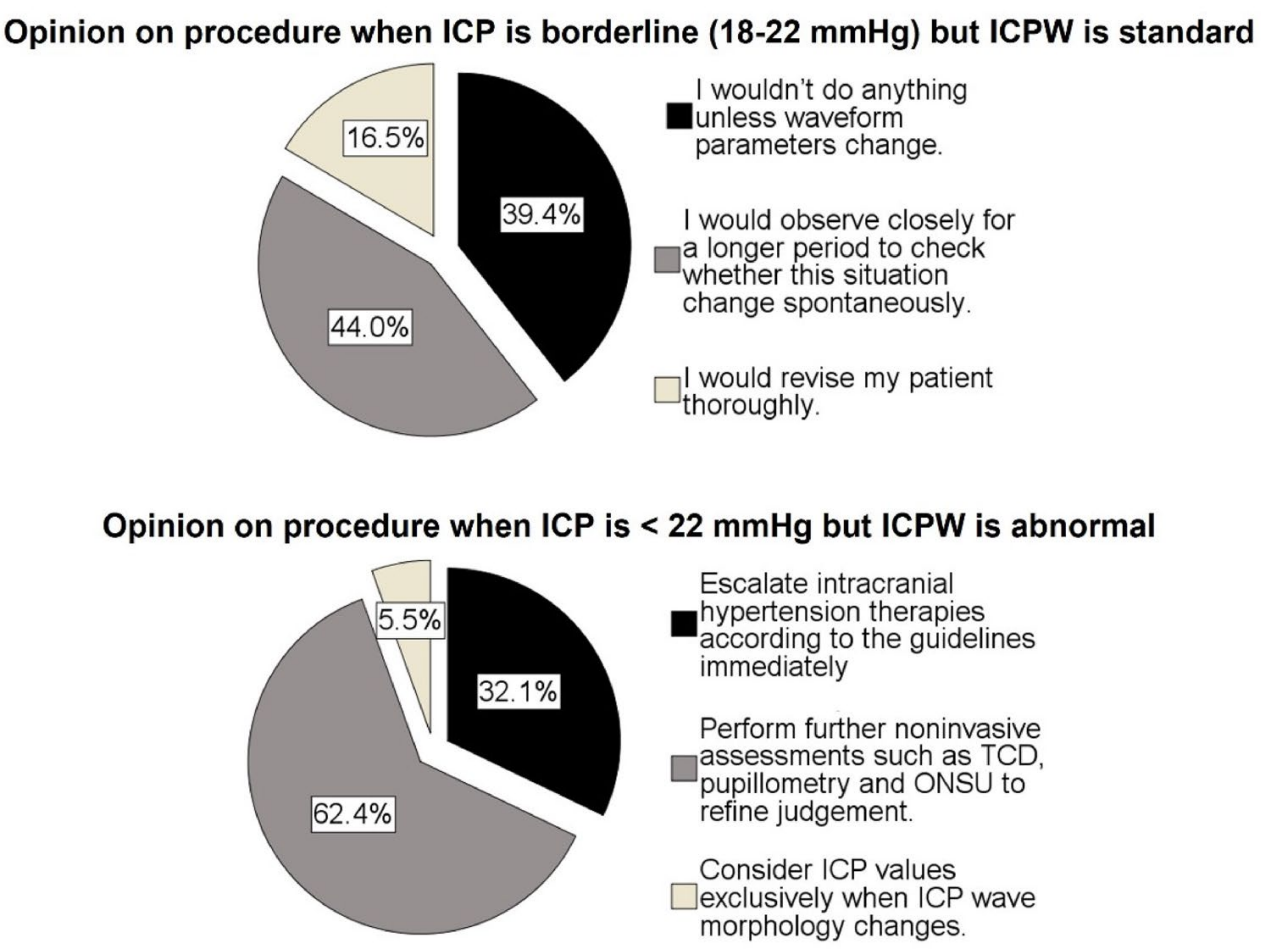
Participants’ answers distributions regarding intracranial pressure waveform (ICPW) relevance according to different intracranial pressure (ICP) levels

Table 1 and Fig. 3 show the responses for each of the survey’s questions. In controversial situations when ICP is under 22 mm Hg but ICPW presents an abnormal morphology suggestive of poor ICC, 84 (77%) participants would review their patients holistically and consider escalating IH TILs between lower tiers 1 or 2. A total of 84 (77%) participants also agreed that a useful way to clarify these situations might be using NIM. The situation of a patient with borderline ICP values between 18 and 22 mm Hg compels 47 (43%) responders to continue just observing when ICPW keeps its normal shape, whereas 62 (57%) would proceed with reassessing the patient and/or taking additional tests to consider escalating lower TILs. Finally, for patients with ICP between 22 and 25 mm Hg but normal ICPW, 33 (30%) participants escalate TILs, 7 (6%) monitor the ICPW to take actions, and 69 (64%) would still proceed to guide actions after performing further ancillary assessments.

**Table 1:** Answers for the survey’s questions

Question	Frequency	Percent (CI 95%)
Adherence to recommendation on escalating ICP interventions when it is ≥ 22 mm Hg		
Do not agree at all, this threshold is not accurate to me	11	10.1 (5.2–17.3)
I partially agree, I am more flexible with thresholds	68	62.4 (52.6–71.5)
I strongly agree	30	27.5 (19.4–36.9)
Agreement on keeping CPP range 60–70 mm Hg		
Do not agree with this range, I always look for an individualized CPP	26	23.8 (16.2–33.0)
I partially agree. I look for this range but only with ancillary support	44	40.4 (31.1–50.2)
I strongly agree. I look for this range on the bedside monitor	39	35.8 (26.8–45.5)
Use of noninvasive ancillary tests to support ICP assessment		
Do not agree at all	4	3.7 (1.0–9.1)
I partially agree. I seldomly check on ancillary tests regarding ICP monitoring	23	21.1 (13.9–30.0)
I strongly agree	82	75.2 (66.0–83.0)
ICPW education during training		
I have no knowledge on this subject	2	1.8 (0.2–6.5)
I did not learn about this subject in residency, I did it by myself	48	44.1 (34.5–53.9)
Yes, I have been trained on this subject very well during my specialization	59	54.1 (44.3–63.7)
Confidence on interpreting ICPW		
I have little knowledge about this subject	3	2.8 (0.6–7.8)
Not much confident. I need to gain a better understanding of the significance of the distinct peaks in the ICP waveform	23	21.1 (13.9–30.0)
Very confident. I know the physiology of ICP wave morphology very well	83	76.1 (67.0–83.8)
ICPW relevance		
It has some importance to me; I check on this in selected cases only	38	34.9 (26.0–44.6)
It is fundamental to individualize my patients’ management	71	65.1 (55.4–74.0)
Perception of difficulty on applying ICPW in clinical practice		
I lack confidence in assessing ICP waveforms visually due to the inherent subjectivity in the process	13	12.0 (6.5–19.5)
I have difficulties identifying the wave components very often	14	12.8 (7.2–20.6)
No, I feel confident about observing and interpreting the ICP slopes	82	75.2 (66.0–83.0)
Familiarity to ICPW parameters (P2/P1 ratio and TTP)		
I don’t know these parameters	9	8.2 (3.8–15.1)
I know a little about this	27	24.8 (17.0–34.0)
Yes	73	67.0 (57.3–75.7)
Support the inclusion of ICPW parameters interpretation to refine ICP management		
Partially agree. Although it is promising information, there still need for more large-scale studies	38	34.9 (26.0–44.6)
Strongly agree	71	65.1 (55.4–74.0)

*CI* confidence interval, *CPP* cerebral perfusion pressure, *ICP* intracranial pressure, *ICPW* ICP waveform, *P2/P1* quotient between second and first ICP waveform peaks, *TTP* time to peak

### Diversity Between LMICs and HICs

Significant differences were observed on ICP management perceptions and practices between LMICs and HICs, as shown in Table 2. Participants from LMICs are more likely to level ABP at the heart than the tragus and to use multimodal NIM as ancillary information to ICP values. Furthermore, participants from LMICs consider ICPW relevant and are more willing to support its implementation in the guidelines as an ICP refinement. Participants who supported updating guidelines toward a personalized ICP management also agreed with adding NIM in this setting (Table 3). There were no differences between LMICs and HICs regarding the use of imaging and transcranial Doppler (TCD) or transcranial color-coded duplex, and these techniques were the most used NIM (70% and 65%, respectively). However, application of optic nerve sheath ultrasound (ONSUS) is significantly higher in LMICs (80%) than in HICs (40.7%, *p* < 0.001). Overall, 71 (65%) participants supported considering the inclusion of ICPW analysis in future guidelines update, whereas 38 (35%) think evidence in this regard is still lacking. Support was significantly more prevalent among LMIC participants (*p* = 0.004).

**Table 2:** LMIC and HIC participants responses on ICP/CPP practices

Question	Total	Country		*p* value
		LMIC	HIC	
Level of ABP measurement				0.003
Heart	60 (55,0)	38 (69,1)	22 (40,7)	
Tragus	36 (33,0)	10 (18,2)	26 (48,1)	
Both	13 (11,9)	7 (12,7)	6 (11,1)	
Agreement to use noninvasive ancillary tests to enhance clinical judgment				0.041
Do not agree at all	4 (3,7)	1 (1,8)	3 (5,6)	
Partially agree	23 (21,1)	8 (14,5)	15 (27,8)	
Strongly agree	82 (75,2)	46 (83,6)	36 (66,7)	
Noninvasive ancillary tests				
Imaging	77 (70,6)	43 (78,2)	34 (63,0)	0.081
TCD	72 (66,1)	39 (70,9)	33 (61,1)	0.280
ONSD	66 (60,6)	44 (80,0)	22 (40,7)	< 0.001
Pupillometry	45 (41,3)	23 (41,8)	22 (40,7)	0.909
Noninvasive ICPW (B4C)	30 (27,5)	25 (45,5)	5 (9,3)	< 0.001
ICPW education during training				0.030
No knowledge on this subject	2 (1,8)	2 (3,6)	0 (0,0)	
Self-taught	48 (44,0)	18 (32,7)	30 (55,6)	
Trained during residency	59 (54,1)	35 (63,6)	24 (44,4)	
Confidence on interpreting ICPW				0.006
Little knowledge/Not much confident	26 (23,9)	7 (12,7)	19 (35,2)	
Very confident	83 (76,1)	48 (87,3)	35 (64,8)	
ICPW morphology relevance				< 0.001
Some importance (selected cases)	38 (34,9)	10 (18,2)	28 (51,9)	
Fundamental to individualize management	71 (65,1)	45 (81,8)	26 (48,1)	
Familiarity to ICPW parameters (P2/P1 ratio and TTP)				0.021
Do not know	9 (8,3)	3 (5,5)	6 (11,1)	
Little knowledge	27 (24,8)	9 (16,4)	18 (33,3)	
Familiar	73 (67,0)	43 (78,2)	30 (55,6)	
Support the inclusion of ICPW parameters interpretation to refine ICP management				0.004
Partially agree	38 (34,9)	12 (21,8)	26 (48,1)	
Strongly agree	71 (65,1)	43 (78,2)	28 (51,9)	

*ABP* arterial blood pressure, *B4C* Brain4Care, *CPP* cerebral perfusion pressure, *HIC* high-income country, *ICP* intracranial pressure, *ICPW* ICP waveform, *LMIC* low/middle-income country, *NIM* noninvasive monitoring, *P2/P1* quotient between second and first ICP waveform peaks, *TCD* transcranial Doppler, *TTP* time-to-peak

**Table 3:** Agreement perception on using NIM and ICP guidelines update

Agreement to use NIM tests to enhance clinical discernment	Agreement level with guidelines updating using ICP plus additional NIM information		Total
	Partially agree	Strongly agree	
Do not agree at all	3 (7,9)	1 (1,4)	4 (3,7)
I partially agree. I seldomly check on ancillary tests regarding ICP monitoring	11 (28,9)	12 (16,9)	23 (21,1)
I strongly agree	24 (63,2)	58 (81,7)	82 (75,2)

*ICP* intracranial pressure, *NIM* noninvasive monitoring

*p* value = 0.019

## Discussion

The current study compiled insights from numerous actively engaged academics with more than a decade of experience in treating neurocritical patients and managing ICP. Participants were divided based on regional economic conditions, distinguishing between LMICs and HICs. This differentiation is particularly significant for assessing the impact of resource availability on medical practices. The collected opinions revealed that practitioners are becoming less rigid in adhering to ICP and CPP thresholds, instead using ICPW and NIM as supplementary tools to inform management decisions. There is a growing confidence in customizing thresholds, such as “optimal CPP” or “critical ICP,” based on the observed autoregulatory status [24].

Novel methods for using ICPW and NIM to increase understanding of cerebrospinal compensatory reserve or brain compliance are emerging [25]. Furthermore, interest in NIM is also as a means to reduce the financial burden related to ICP monitoring, which has grown exponentially in the last few decades [26]. Understanding of ICPW and its clinical application receives attention whether professionals are from LMICs or HICs and its interpretation seems to be determinant on decisions. The participants of the present survey agreed with the addition of these components to be considered in future guideline revisions.

**Fig. 4:**
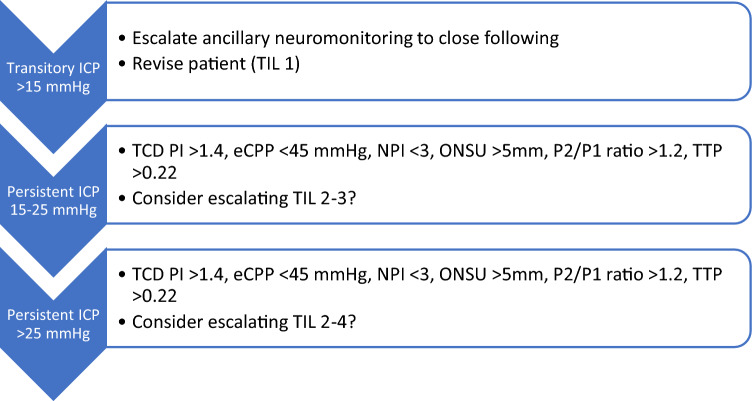
A flowchart proposal (still to be prospectively validated) for ICP management, considering evidence-based ancillary noninvasive neuromonitoring. Techniques may include transcranial Doppler, pupillometry, ICP waveform, and ONSUS. *eCPP* estimated cerebral perfusion pressure, *ICP* intracranial pressure, *NPI* neuropupillary index, *ONSUS* optic nerve sheath ultrasound, *P2/P1* quotient between second and first ICP waveform peaks, *TIL* therapy intensity level, *TTP* time to peak

With reference to the most cited NIM techniques by the surveyed, TCD has been acknowledged by its capacity of assessing blood velocities and observe CPP reduction by means of the pulsatility index [27] or even the ICP/CPP estimation [28, 29]. Furthermore, TCD is highly sensitive to cerebrovascular dynamics, allowing clinicians to assess immediate responses at the bedside in the case of changes in ABP and pCO_2_, hydrocephalus, midline shift, and brain death (e.g., see [30]). The ONSUS has been extensively studied in the last years, with a threshold of ~ 5.8 mm currently adopted as a suitable cutoff for elevated ICP [31]. Among ONSUS advantages are low costs, short learning curve, and readiness. The pupil-reactivity index (NPi), derived from automated pupillometry, has been the most studied parameter of this technique for its correlation with ICP, understanding NPi decrease as a potential indicator of ICP elevation [32], while one multicentric trial observed significant correlation between persistent NPi < 3 and poorer outcomes in neurocritical care [33]. One emerging technique cited by 27.5% of the surveyed (Table 2) reproduces ICPW following pulsatile micrometric dilations of the skull at each heartbeat. Its generated waveforms undergo automated analysis and numerical ratios as the quotient between second and first ICP waveform peaks (P2/P1) and time to peak may aid physicians detecting poor ICC [34]. Figure 4 summarizes this combination of NIM with their respective indicators. Studying such a model and how it can aid in improving ICP judgment warrants prospective validation.

### CPP

Cerebral perfusion pressure is the force with which blood penetrates brain tissue and its adequacy is fundamental to meet the brain's metabolic demand [20]. It was described more than 60 years ago by Lassen [35], who hypothesized that CPP would be the difference between the input (mean ABP [MAP]) and the intracranial resistance provided by the brain tissue and the venous outflow, or ICP. These concepts were proven to be true among healthy individuals, and the formula CPP = MAP − ICP is currently adopted in clinical practice and widely present in neurocritical care literature [36]. The Brain Trauma Foundation recommend the 60–70 mm Hg range [37], whereas the Lund recommendations are to keep CPP near 50 mm Hg in severe TBI [38]. However, once either chronic or acute situations that impair cerebral autoregulation are present [39, 40], CPP manipulation strictly by means of changes in ABP just to compensate ICP may be iatrogenic [41].

The extremely rich branching of cerebral vessels creates a progressive reduction in the actual ABP in intracranial vessels from the circle of Willis to the cerebral small vessels [42]. This lower ABP, associated with absence of muscular wall in the final arterial line make these vessels much more sensitive to higher ICP and easily collapsible after acute brain injuries, expanding hypoperfusion to its surrounding areas [43]. Therefore, cerebral blood flow (CBF) stops in the small vessels even with ABP being far from zero, the so-called critical closing pressure which changes in strict adherence with ICP fluctuations [44]. Furthermore, it was demonstrated that the counterforces between MAP and ICP do not behave as balanced as the formula preconizes, being the cerebrovascular capacity to compensate CPP in face of ICP elevations much more mitigated when compared to cerebrovascular response to ABP changes [45, 46].

**Fig. 5:**
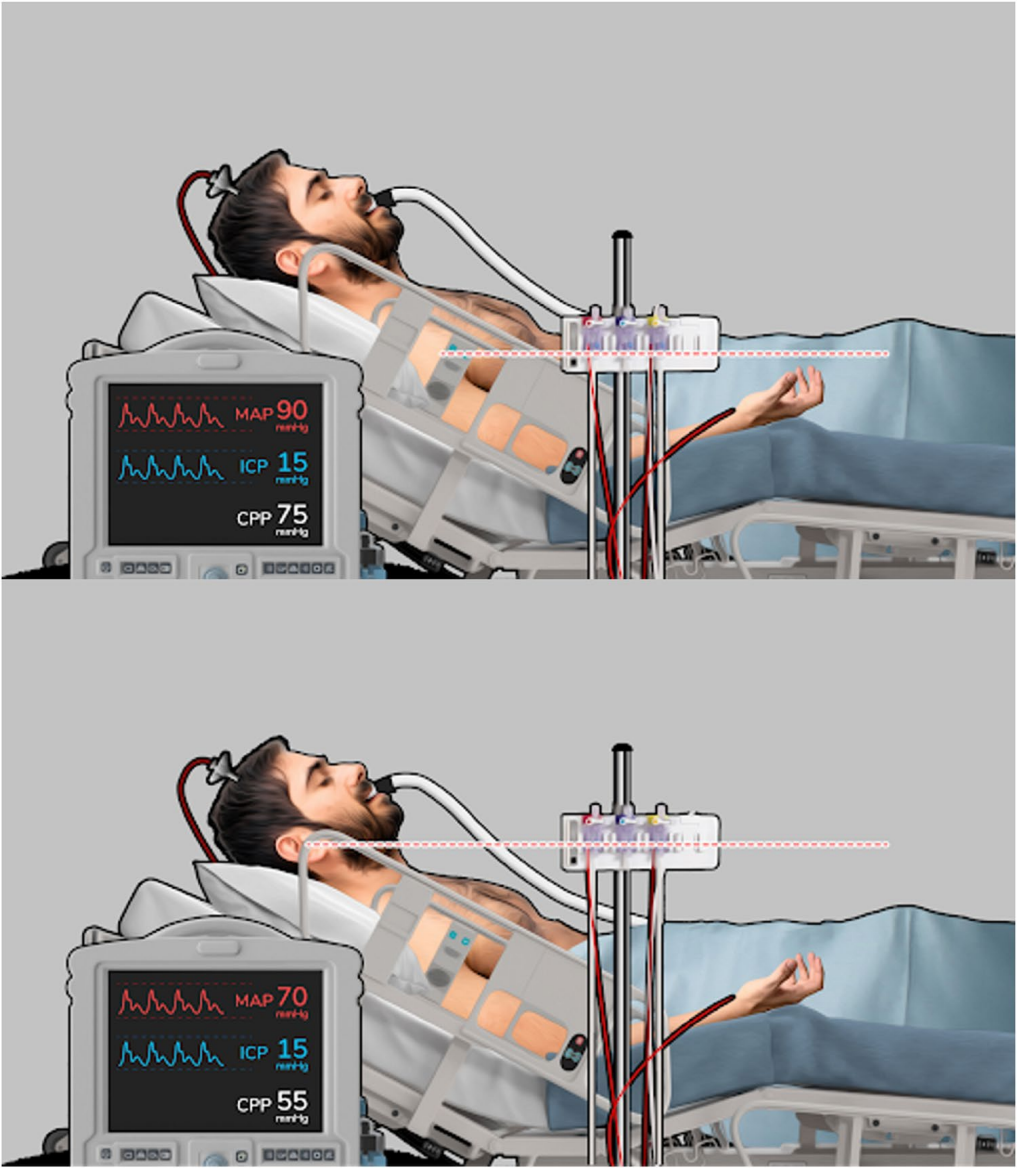
A representation of how changing ABP level from right atrium to tragus may change CPP calculation. Top, CPP is overestimated in 75 mm Hg, whereas real CPP is 55 mm Hg in the lower picture. It is fundamental to consider patient positioning (bed and head angle) and that these CPP values differences vary among individuals. *ABP* arterial blood pressure, *CPP* cerebral perfusion pressure

With all the above, it may be concluded that assessing CPP as the difference between MAP and ICP may not be accurate. Several efforts are being made to provide an accurate estimation and optimization of CPP [47, 48]. Unfortunately, optimal CPP is still under development [49, 50] and needs dedicated systems that are not widely available especially in LMICs. To estimate CPP properly, it is recommended to monitor ABP with the transducer leveled at the tragus [36, 51, 52], but our result in this regard indicate that this is not always the case, which is consistent with a previous survey among the Brain Trauma Foundation authors [53]. An ABP leveled at the heart may overestimate CPP between 10 to 20 mm Hg [36, 54] (Fig. 5). However, even with ABP leveled at the tragus, the resultant CPP may not represent the whole brain CPP. When available, TCD [29] and brain oximetry [55] provide adjunctive information.

**Fig. 6:**
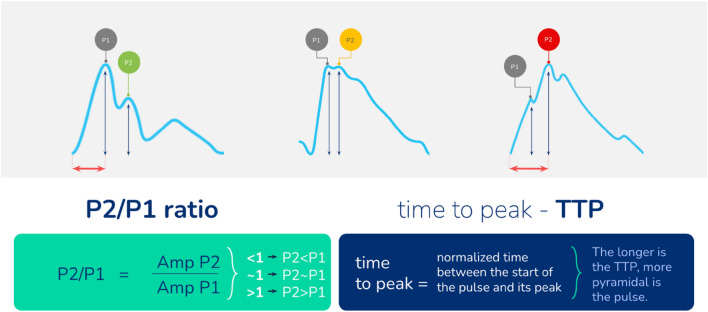
Parameters extracted and calculated from intracranial pulse slopes. P2/P1 ratio is the ratio between second and first peak amplitudes, whereas TTP is the time from pulse upstroke to the highest amplitude identified, represented in percentage. Rounded intracranial pressure pulse shapes indicating reduced intracranial compliance present with higher P2/P1 ratios and TTP

### Adherence to ICP Management Guidelines

The Seattle International Severe Brain Injury Consensus Conference recommended that physicians use their clinical judgment and adapt the guidelines as best possible [6]. In the present survey, 70% of participants described their practice as flexible with thresholds; this was independent of being based in a LMIC or HIC. LMIC participants were more favorable in considering the inclusion of additional information such as ICPW parameters and NIM in determining IH actions. Probably the most suitable explanation for this finding is the lack of invasive ICP for all patients in need in LMIC areas, compelling these physicians to build up experience using NIM.

In healthy adults, ICP values remain under 15 mm Hg [51, 56]. In face of a sustained range of 22–25 mm Hg, 30% of this survey’s participants opt to escalate IH TILs regardless of any further information. For borderline ICP values like 18–22 mm Hg (and possibly lower values in selected cases) the need for additional data becomes evident, since allowing ICP to remain beyond individual safety thresholds may carry inadvertent consequences [56]. This is supported by the recent work from Riparbelli et al. [4], in which a cohort of more than 300 patients found 18 mm Hg as a threshold associated with mortality and unfavorable outcomes.

### ICPW

Intracranial pressure pulse morphology carries useful information [15, 21], ancillary to the mean ICP typically shown on bedside monitors (which is measured by averages of time intervals) [57]. The changes in beat-by-beat ICPW have been extensively studied, especially the correlation between its different peak amplitudes (P1, P2, and P3) following changes in the volume/pressure relationship [58–60]. Inside the bony box of the skull, the continuously moving interaction between arterial blood flow with the brain and ventricles filled with cerebrospinal fluid, determines the formation of P1 (upstroke peak), P2 (tidal wave), which is associated with ICC, and the buffering reserve. Finally, P3 is associated with the closure of the aortic valve [58]. Because ICP is correlated with CBF, other variables that influence CBF may also influence ICPW, such as the ventilatory status [61], blood viscosity, temperature, and the cardiac output [62].

ICPW parameters including P2 elevation may precede an IH crisis by several minutes [17, 18]. This observation led to an increased interest in exploring the parameter P2/P1 ratio, as a descriptor of decreased adaptative capacity [63, 64]. Brasil et al. [16] found the P2/P1 ratio to increase around 10% after promoting an ICP increase of ~ 4 mm Hg from a baseline of ~ 15 mm Hg in a study of patients with TBI without skull damage. Integrated artificial intelligence–based pulse shape index tracing continuously P2 to P1 proportions has recently been proven to associate both with outcome after TBI and CT findings [65, 66]. The time to peak (TTP) is a normalized parameter derived from each pulse triggering up to its highest amplitude [67, 68]. The pulse slope triggering is time zero and the end of the pulse is time hundred, therefore TTP is the percentage representation of the pulse’s length in which the highest amplitude is identified (Fig. 6). These parameters, currently available exclusively in a noninvasive device [34] soon will be also included in invasive systems to gather ICP values and ICPW automated metrics [69], what will aid practitioners on interpreting ICPW changes instead of making a simple visual assessment. Given the multiple inherent variables included in this phenomenon [57], distinguishing the distinct ICPW peaks without the support of a dedicated analytics process may become difficult.

In this survey, it was noted that as ICP exceeds 22 mm Hg, fewer experts rely on ICPW for decision-making. However, ICPW garners more attention in distinguishing patients below this threshold but at risk of experiencing ICC impairment. Therefore, the accurate use of ICPW hinges on the clinician’s confidence in interpreting its patterns. The vast majority of participants expressed confidence in interpreting and applying ICPW in their clinical practices, citing their understanding of the pathophysiology of patients with severe neurological injury.

### Limitations

Limitations of the present study include relying on the limited area of survey distribution, which included primarily professionals from Europe and North, Central, and South America, having no participants from Oceania, Asia, and Africa. Survey responses were kept anonymous; therefore, the accuracy of the information provided and fulfillment of prerequisites for participating was based on good faith. The survey did not embrace questioning participants on their perceptions regarding ICP levels more than 25 mm Hg, although it is implicit that over this level, professionals take their actions to treat IH independently of any further information. The survey considered LMICs and HICs exclusively, according to the real situation of their health services, and therefore the results may not represent the actual heterogeneity of resources available especially in LMIC.

## Conclusions

Experienced professionals are prone to the individualization of ICP thresholds, by means of a variety of ancillary parameters, which can be obtained using other invasive or noninvasive techniques. Among a majority of these professionals, ICP pulse morphology and NIM techniques (imaging, TCD, pupillometry, and ONSUS) were considered valuable options to be included in future guideline updates. Especially when ICP is below 22 mm Hg but ICC seems to be compromised, the use of supportive invasive or noninvasive techniques to refine bedside judgment increases. Professionals from LMICs are likely more supportive of these ancillary methods to alter IH TILs. Nevertheless, prospective studies to create the ideal ICP monitoring and treatment algorithm are still needed. These implications are fundamental to assess CPP, which also currently lacks standardization in its measurement.

## Supplementary Information

Supplementary file 1
